# Mechanical Properties of Poly(Alkenoate) Cement Modified with Propolis as an Antiseptic

**DOI:** 10.3390/polym15071676

**Published:** 2023-03-28

**Authors:** David Alejandro Aguilar-Perez, Cindy Maria Urbina-Mendez, Beatriz Maldonado-Gallegos, Omar de Jesus Castillo-Cruz, Fernando Javier Aguilar-Ayala, Martha Gabriela Chuc-Gamboa, Rossana Faride Vargas-Coronado, Juan Valerio Cauich-Rodriguez

**Affiliations:** 1 Facultad de Odontologia, Universidad Autonoma de Yucatan, Calle 61-A x Av., Itzaes Costado Sur “Parque de la Paz”, Col. Centro, Merida 97000, Yucatan, Mexico; david.aguilar@correo.uady.mx (D.A.A.-P.);; 2 Centro de Investigacion Cientifica de Yucatan A.C, Calle 43 # 130 x 32 y 34, Colonia Chuburna de Hidalgo, Merida 97205, Yucatan, Mexico

**Keywords:** bioactive materials, antibacterial properties, antibiofilm activity

## Abstract

Background: We assessed the effect of propolis on the antibacterial, mechanical, and adhesive properties of a commercial poly(alkenoate) cement. Methods: The cement was modified with various concentrations of propolis, and antibacterial assays were performed against *S*. *mutans* by both MTT assays and agar diffusion tests. The compressive, flexural, and adhesive properties were also evaluated. Results: the modified cement showed activity against *S*. *mutans* in both assays, although reductions in compressive (from 211.21 to 59.3 MPa) and flexural strength (from 11.1 to 6.2 MPa) were noted with the addition of propolis, while adhesive strength (shear bond strength and a novel pull-out method) showed a statistical difference (*p* < 0.05). Conclusion: the antiseptic potential of modified material against *S*. *mutans* will allow this material to be used in cases in which low mechanical resistance is required (in addition to its anti-inflammatory properties) when using atraumatic restorative techniques, especially in deep cavities.

## 1. Introduction

Poly(alkenoate) cement (PAC) is a material that contains calcium fluoroaluminosilicate glass and an aqueous solution of poly(acrylic acid) (PAA) in the original formulation and is commonly used as a base or restorative material in dentistry [1]. High-viscosity PAC is recommended for atraumatic restorative techniques (ARTs) [2], but their applications have been expanded for bone cement replacement when properly formulated [3,4]. Some of their benefits include low pulp inflammation, chemical adhesion to enamel and dentine, low shrinkage, a similar coefficient of thermal expansion to dental tissue, sustained fluoride release, good resistance to marginal filtration, and acceptable mechanical properties [5].

Despite all these advantages, earlier improvements in the original formulation were due to moisture sensitivity and being prone to incomplete crosslinking in the presence of unionized PAA, being especially relevant in PAC as luting cement, as they contain more PAA, which inhibits the formation of apatite interlayers, which are essentials to marginal gap sealing [6]. Therefore, to palliate these undesirable properties and to enhance its properties, the use of additives (i.e., tartaric acid for stronger aluminum complex formation and extending working times) [7], polyelectrolytes based on the copolymers of acrylic acid and unsaturated acids (maleic or itaconic acid for more rigid crosslinking with aluminum) and the inclusion of metals, such as silver and tin, has been proposed [8]. Recently, other types of improvements have considered the use of different fillers and novel compositions to enhance mechanical performance [9].

The mechanical performance of PAC is assessed by both compressive and bending behavior, which are generally defined by international standards despite the fact that the material in oral cavities is subject to complex loads [10,11]. Thus, the strength values measured by using international standards do not represent the mechanical conditions necessary for clinical uses [12]. Therefore, the reported mechanical properties of commercial dental materials vary widely, and generally, when a new formulation of PAC is developed, it is common to consider that higher mechanical properties are desirable [13].

Another important parameter for dental material performance is the adhesion to either the enamel or dentine in the tooth; conventional PAC bonding to dentine, which contains more water while being less mineralized, is more difficult compared to enamel, but due to its hydrophilic nature, it can wet the surface and provide adequate bonding, especially after removing the smear layer with weak (ascorbic) or strong (phosphoric) acids [14].

Many efforts were made to improve adhesion, and a wide variety of tests have been proposed to determine this property among the restorative material and the tooth surface as the shear [13,15], tension [16], and push-out bond strength [17]. Other mechanical methods were proposed in the ISO/TS 11405 standard (dentistry-testing of adhesion to tooth structure) [18] and ISO 29022 standard (dentistry-adhesion-notched edge shear bond strength test) [19].

Fluoride release is another important issue regarding PAC, not only because of its remineralization potential on recurrent caries, but also because it inhibits plaque formation, with claimed antibacterial action [20]. However, this is not enough to overcome secondary caries, and several additives have been used, including chlorhexidine (CHX), quaternary ammonium salts (QAS), metallic particles, modified polymers, etc. [21,22,23,24]. Propolis, a natural product with well-documented antibacterial and antimycotic properties, has also been suggested as an additive to dental materials [25,26,27,28]. Other natural extracts that have been used include green tea, *Triphala* (an ayurvedic herbal formulation that contains three medicinal plants: *T*. *chebula*, *T*. *belerica*, and *Phyllanthus embelica*), *Salvadora persica*, *Olea europaea,* and *Ficus carcia*, which has been proven to have numerous benefits [29,30,31].

Propolis-modified dental materials can accelerate wound healing due to anti-inflammatory responses, as tissue repair is mediated by their inflammatory mechanism [32]. Furthermore, controversial results exist on propolis addition to PAC; for example, microhardness has been reported to increase via the use of the ethanolic extracts of propolis [33], whereas a reduction in compression strength has been reported due to adding propolis to PAC type II [34], but with an increase in compressive strength regarding high-viscosity PAC [35]. However, in these studies, little emphasis was put on the changes in terms of tooth adhesion. Therefore, the aim of this study was to assess the effect of propolis on *Streptococcus mutans* viability, identified as the most common micro-organism associated with the initial phase of caries [36]. In addition, the mechanical properties of compression, flexion, and adhesion (newly proposed pull-out test) were studied.

## 2. Materials and Methods

### 2.1. Materials

Ethanolic solution of propolis (20 wt%, Brand Yucamiel) with a total flavonoid content of 25.94 ± 2.06 mg of quercitin/g, phenol content of 49.68 ± 0.29 mg garlic acid/g, 2.5 µg/mL of average inhibitory concentration (IC50), and antiradical power (1/IC50) of 0.40 was used for all experiments, as reported in a prior publication [32]. Fuji IX PAC was purchased from GC Corporation (Tokyo, Japan), while *S*. *mutans* was acquired from ATCC (25175).

### 2.2. Preparation of PAC and Modified PAC (MPAC)

Both materials, PAC and MPAC (formulations described in Table 1), were prepared according to the manufacturer’s specifications by hand mixing the powder and aqueous solution of PAA (3.6:1, respectively) and then adding a specific volume of 20 wt% ethanolic solutions of propolis [27,28,33]. After the final mixing, the paste was placed in molds with the corresponding geometry for each test.

### 2.3. Physicochemical Characterization of PAC and MPAC

Fourier-transform infrared (FTIR) spectra were obtained using a Nicolet 8700 spectrometer in the spectral range between 4000 and 400 cm^−1^ using KBr pellets. X-ray diffraction (XRD) patterns were obtained using a Siemens D-5000 Bragg diffractometer in the 2θ range from 10° to 60°, with an interval time lapse of 4 s and a step size of 0.02°.

### 2.4. Microbiological Test

Both materials, PAC and MPAC (12.5 µL, 25 µL, and 50 µL of propolis), were prepared in a silicon mold with a 6.35 mm diameter and 2 mm in thickness. Then, each disc was immersed in 3 mL of sterile distilled water for 24 h at 37 °C. Finally, 100 µL of the obtained eluates were placed in a 96-well plate along with 100 µL of the *S*. *mutans* inoculum. As a negative control, amikacin (1 mg/mL) was used, while only bacteria were used as the positive control. Bacteria viability was determined using 100 µL of MTT after incubation for 24 h at 37 °C. Additionally, the antibacterial activity of MPAC against *S*. *mutans* was also assessed by the agar diffusion method; discs of the same dimension (as mentioned above) were placed over bacterial seeding of brain heart infusion (BHI) agar and then placed in an incubator at 37 °C for 24 h. A paper soaked in amikacin was used as the negative control, and a disc without propolis was used as the positive control.

### 2.5. Mechanical Characterization

#### 2.5.1. Compressive

Tests were conducted according to the ISO 9917-1 standard, using cylindrical samples 6 mm in height and 4 mm in diameter that were obtained using a Teflon mold.

#### 2.5.2. Three-Point Bending

Tests on PAC and propolis-modified MPAC were conducted according to ISO 9917-2. For this, rectangular specimens (25 mm × 5 mm × 1.75 mm) were obtained after Teflon mold casting. Thus, the samples were stored in distilled water at 37 °C for 24 h according to the standard.

Both mechanical tests were conducted on samples containing 25 µL and 50 µL of propolis and carried out in a Shimadzu AGS–X (Kyoto, Japan) universal testing machine with a 5 kN and 1 kN load cell, respectively, and a crosshead speed of 1 mm/min. Five samples were used by the group, then the mean and standard deviation were reported.

#### 2.5.3. Shear Bond Test

**Figure 1:**
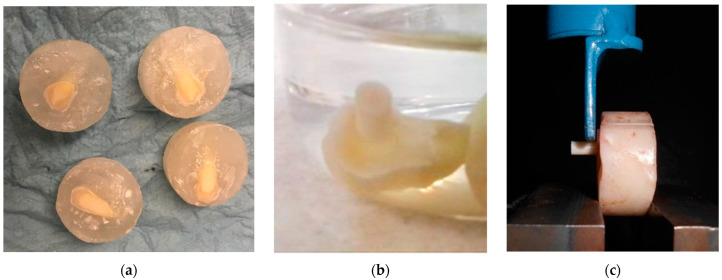
Preparation of samples for shear adhesive test. (**a**) Dentin after polishing. (**b**) MPAC cured on dentin. (**c**) Shear adhesion test.

Adhesion to dentine was assessed by shear bond testing, suggested by the ISO 29022 standard and by other authors [37,38]; molars were polished with silicon carbide (number 400) abrasive paper until the dentin was exposed, Figure 1a, with an average area of 5 mm^2^; then, conditioning the tissue with poly(acrylic acid) for 15 s was carried out, and then a cylinder was cured over the treated surface (MPAC-A). To understand the effect of the propolis, shear adhesive behavior was assessed after four different treatments. For the first case, dentin was propolis-treated with no acid, and then PAC was cured on the surface, referred to as PAC-NA. For the second case, the exposed dentin was treated with propolis and then 1 mL of poly(acrylic acid), instead of the conventional 32–37% phosphoric acid, for 15 s, and then a PAC cylinder was cured over the treated surface (referred as PAC-A). In addition, two controls were manufactured as follows: (a) the first control was prepared with PAC without propolis, while dentin was treated with poly(acrylic acid), denoted as PAC-A, and (b) PAC was cured on dentine without propolis treatment and without poly(acrylic acid) treatment, referred to as PAC-NA. All samples described above were stored in distilled water, Figure 1b, for 24 h at 37 °C according to the standard. The test was carried out with a Shimadzu AGS-X (Kyoto, Japan) universal testing machine, with a 100 N load cell and crosshead speed of 1 mm/min, Figure 1c; five samples were tested, with the mean and standard deviation reported. After detaching, the samples were gold coated and observed by using a JEOL JMS 6360LV scanning electron microscope with an accelerating voltage of 20 kV.

#### 2.5.4. Pull-Out Test

**Figure 2:**
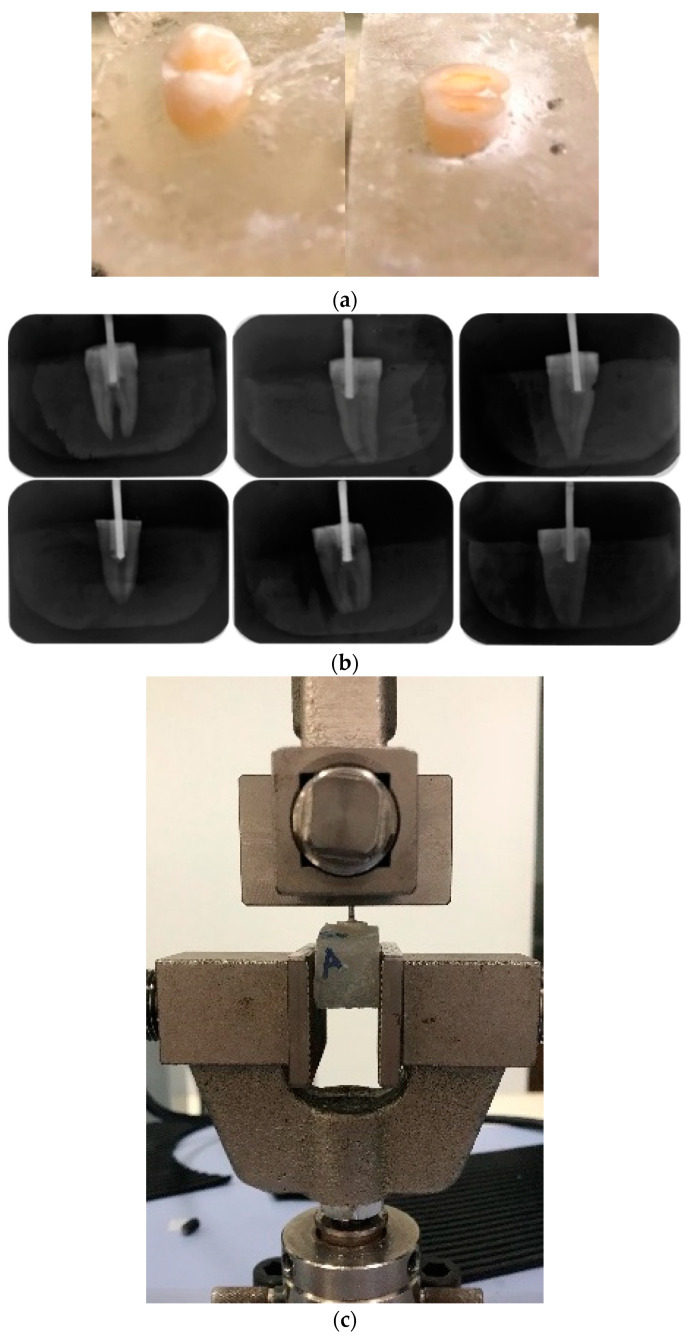
Pull-out test for measuring adhesion strength. (**a**) Premolars embedded in an acrylic resin. (**b**) Radiographs of premolar with metal post. (**c**) Pull-out test.

In addition, a nonstandardized pull-out test was used as a novel method, using the inner surface of the dental tissue treated by different protocols before PAC filling. Groups of 5 premolars per test were embedded vertically in an acrylic resin and then polished until the occlusal portion was removed and a flat surface was obtained (Figure 2a). This flat surface was drilled 6 mm deep while irrigating with distilled water. Strength behavior was assessed prior to the four different treatments; (1) dentin was propolis-treated with no acid (MPAC-NA), then filled with the PAC; (2) the exposed dentin was treated with propolis, then with 1 mL of poly(acrylic acid) for 15 s, and then a PAC cylinder was cured over the treated surface (MPAC-A); (3) PAC without propolis, while the dentin was treated with poly(acrylic acid), denoted as PAC-A, and (4) PAC was cured on dentine without propolis treatment and without poly(acrylic acid) treatment, referred to as PAC-NA. Before the curing process, a metallic root was inserted in the center of the cavity. To verify its alignment, periapical radiographs were obtained, Figure 2b; then, the samples were placed in an incubator in the presence of a saline solution for 24 h at 37 °C. The maximum adhesive force required to remove the complete PAC from the cavity treated was recorded using a Shimadzu AGS-X (Kyoto, Japan) universal testing machine with a 5 kN load cell and a crosshead speed of 1 mm/min, Figure 2c. The mean and standard deviation were reported.

### 2.6. Statistical Analysis

The statistical analyses used were a one-way ANOVA with Bonferroni (posthoc), *p* < 0.05 for the compressive, bending, pull-out, and shear bond strength of PAC and MPAC.

## 3. Results

### 3.1. Physicochemical Characterization

**Figure 3:**
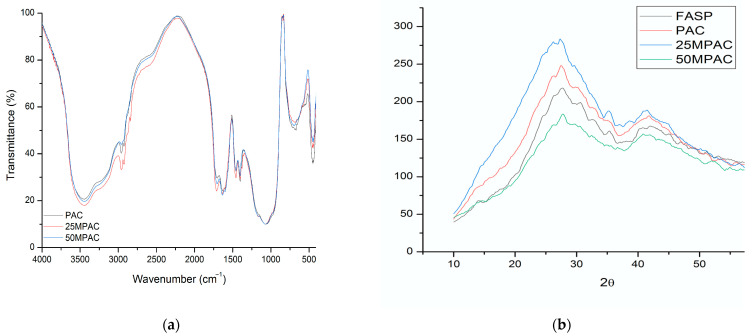
Physicochemical characterization of propolis-modified poly(alkenoate) cements; (**a**) FTIR spectra; (**b**) X-ray diffraction pattern.

The unmodified PAC shows absorptions via FTIR (Figure 3a) at 3436 (OH), 3124, 2965 (CH_2_), 1720 (COOH), 1635 (C=C vinyl), 1602 (COOasym-Al), 1463 (C-H or COOsym), 1401 (COOsym), 1166 (CO of Poly(acrylic) or tartaric acid), 1076 (C-O), 796, and 644 cm^−^^1^. This assignment, however, should consider that, in pure calcium polyacrylates, asymmetric stretch absorptions appear at 1550 cm^−^^1^, which correspond to Al at 1599, with calcium and aluminum tartrate at 1595 and 1670 cm^−^^1^, respectively. Additionally, we note bands at 1410, 1460, 1385, and 1410 cm^−^^1^, corresponding to the symmetric stretching of COO or CH_2_ bending. Thus, the absorptions between 1401 and 1463 can be attributed to the polycarboxylates or tartrates of Ca and Al. Finally, the bands between 400 and 800 cm^−^^1^ could correspond to crystalline structures associated with Al_2_O_3_, metallic fluorides, etc. As depicted in Figure 3a, little modification to the FTIR spectra was observed after propolis addition.

The X-ray diffraction patterns for PAC and MPAC in Figure 3b show that there were no new reflections after propolis modification, i.e., the fluoroaluminosilicate powder was amorphous in addition to the final poly(alkenoate) cement.

### 3.2. Antibacterial Properties

**Figure 4:**
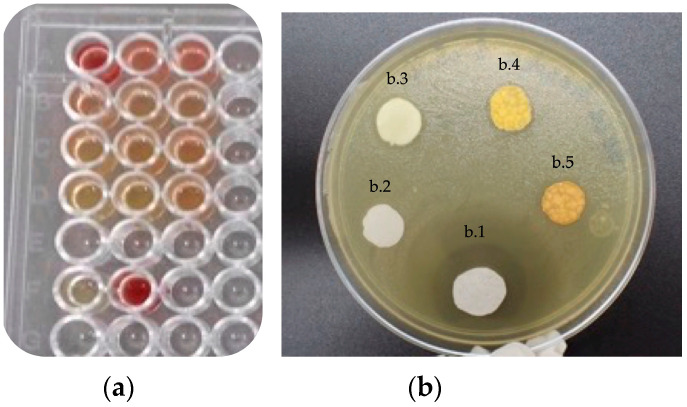
Antibacterial activity of propolis-modified PAC against *Streptoccocus mutans*. (**a**) MTT assay. (**b**) Agar diffusion method; b.1 Amikacin, b.2 negative control, b.3 12.5MPAC, b.4 25MPAC, and b.5 50MPAC.

Figure 4a shows the results of the MTT viability tests, observing that the MPAC extracts can stop *S*. *mutans* growth (see yellow color in the second (B) to fourth (D) row in Figure 4a). In contrast, the extract from PAC did not inhibit bacteria growth (see red color in the first row (A) in Figure 4a). The sixth row (F) showed the effectiveness of the amikacin negative control (yellow, F1) and the only bacteria growth, which is stained in deep red (F2).

Figure 4b shows the inhibition halo in the presence of *S*. *mutans* after contact with the propolis extracts. Amikacin exhibited an inhibition halo of 8 mm (labeled as b.1), while MPAC showed 1.61 mm (labeled as b.3), 1.97 mm (labeled as b.4), and 2.0 mm (labeled as b.5) for 12.5, 25, and 50 µL of propolis in the PAC, respectively, as measured with ImageJ software. No inhibition halo was observed in the PAC-only disc (labeled as b.2).

From these results, it is suggested that MPAC has antiseptic activity against *S*. *mutans* as the MIC of the ethanol solutions of propolis was 2.5 µg/mL.

### 3.3. Mechanical Properties

#### 3.3.1. Compressive Strength

**Figure 5:**
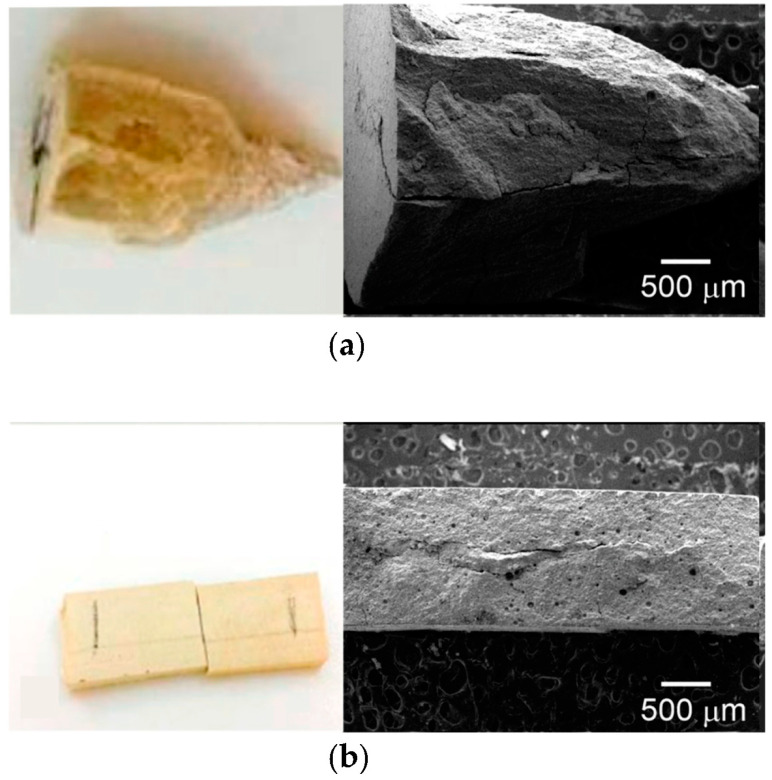
Fracture surface of (**a**) compression and (**b**) bending. Both fracture patterns indicate a rigid material.

Unmodified PAC exhibited a compressive strength of 211.21 MPa, which was reduced by up to 59.36 MPa with 50 µL of propolis. However, the unmodified PAC value is much higher than the other reported values for the same Fuji IX cement, where 152.4 MPa was reported after 24 h [16,39]. According to ISO 9917-1, the compressive strength should be 100 MPa for restorative polyalkenoates and 50 MPa for base/lining cement. It has been established that the masticatory forces can be 91 N (anterior) and 129 N (posterior), with a maximum of 314 N [40]. Overall, these results suggest that, at the highest concentrations of propolis (50 µL), MPAC was not suitable for restoration, but at the lowest concentration of propolis (25 µL), it is on the limit for its use as a liner. From the fracture surface shown in Figure 5a for compression, it seems that the fracture pattern is not modified, and therefore the poor mechanical properties exhibited are due to curing interference by the propolis compounds, probably with chelating properties, and not due to jvcr@cicy.m the presence of ethanol.

#### 3.3.2. Bending Strength

In agreement with this, the bending strength decreased from 11.1 MPa to 6.25 MPa from PAC to MPAC, with the highest concentration of propolis. As mentioned before, the fracture surface pattern was like the unmodified PAC (see Figure 5b). According to the ISO 9917-2 specifications, flexural strength should be no less than 20 MPa for restorative cement and no less than 10 MPa for bases and liners. In this regard, Xie et al. [1] reported Fuji GIC values of 71.1 and 26.1 MPa for Fuji II LC and Fuji II, respectively. In addition, Hu et al. [29] reported 25 MPa of flexural strength for a green tea extract (epigallocatechin-3-gallate)-modified GIC. Therefore, the large amounts of propolis used here (25 µL and 50 µL) do not fulfill the requirements of the standard. The compressive and bending properties are summarized in Table 2.

#### 3.3.3. Shear Bond Strength

PAC that was cured in the presence of 25 µL propolis and then adhered to dentin for shear bond testing (25 MPAC) was used additionally for comparison purposes, as the compressive properties suggest their use as a restorative material. This sample showed an adhesion strength of 1.47 ± 0.21 MPa, while PAC only exhibited 1.02 ± 0.01 MPa on nonacid-treated dentin. This increase has been previously reported for nano-MgO-modified PAC, exhibiting a shear bond strength as high as 5 MPa on the enamel of bovine incisors, which increase up to 6 MPa when tested on dentine [41].

**Figure 6:**
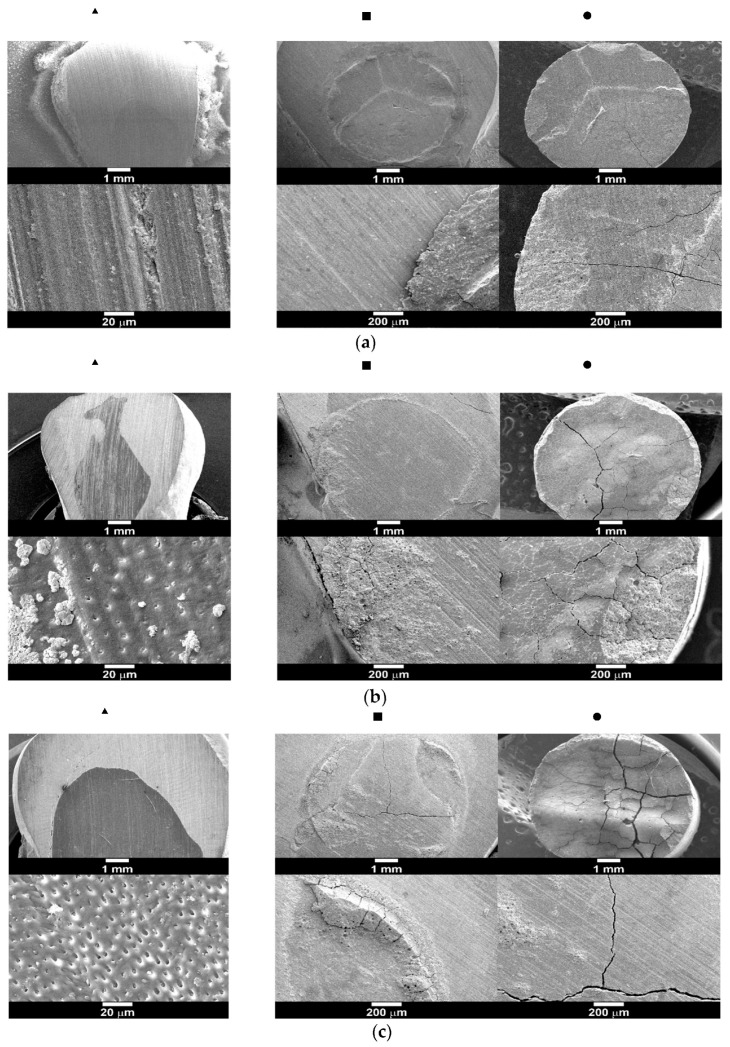
Surface appearance after shear bond testing; PAC-NA (**a**), PAC-A, (**b**), and MPAC-A (**c**). ▴ Tissue appearance after surface treatment (first column); ▪ tissue appearance after detachment (second column); ● cylinder surface after detachment (third column). Top row: low magnification (scale bar 1 mm). Bottom row: high magnification (scale bar: 20 µm or 200 µm).

Propolis-treated dentine (MPAC-NA) cylinders did not adhere to the dentin surface, probably due to the nonpolar components of propolis. However, in non-propolis-treated dentin and nonacid-treated dentin unmodified glass ionomer cement (PAC-NA), the shear bond strength was 0.62 ± 0.27 MPa. When the dentine surface was acid-treated only with no propolis treatment (PAC-A), the shear bond strength increased up to 1.70 ± 0.53 MPa. For the propolis-treated (followed by acid treatment) dentine surface (MPAC-A), the shear bond strength was 1.71 ± 0.74 MPa, i.e., there was no change in the adhesion. The type of failure (adhesive or cohesive) is shown in Figure 6 only for those samples that adhere to the surface, i.e., MPAC-NA is not shown. In Figure 6a, the PAC-NA samples showed adhesive failure predominantly, but in some cases, mixed types of adhesive failure were observed. In Figure 6b, the PAC-A samples suffered a mixed type of adhesive failure. Finally, in Figure 6c, the MPAC-A samples also suffered a mixed type of adhesive failure.

**Figure 7:**
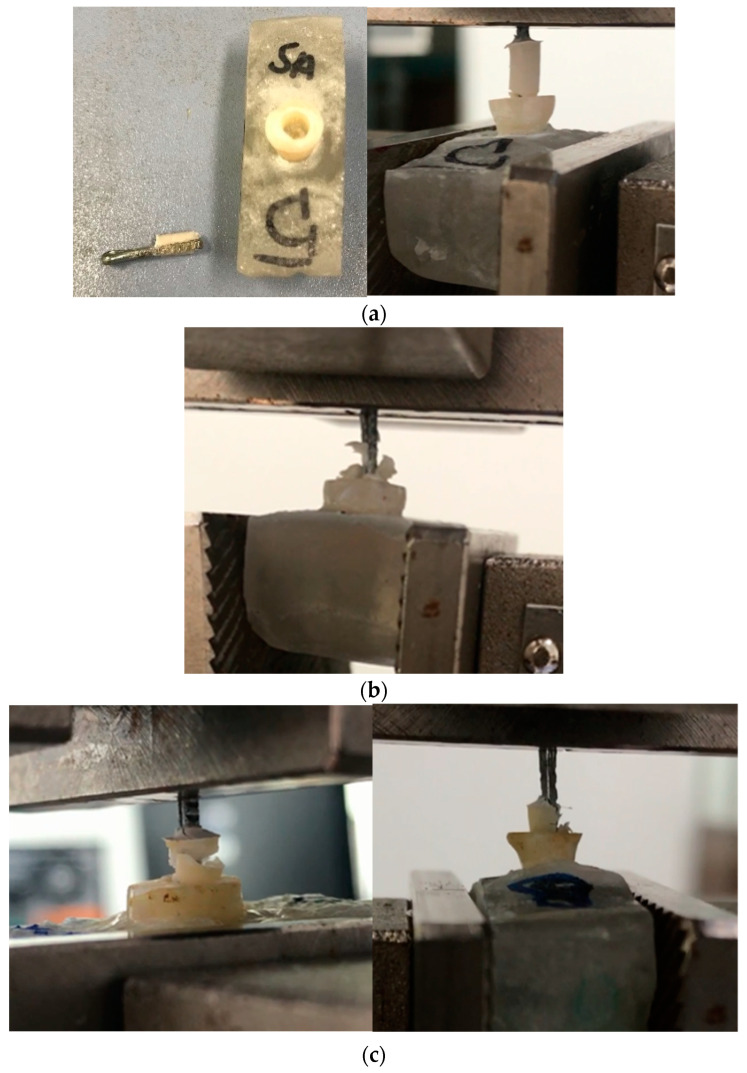
Type of failure after the pull-out test. (**a**) No propolis and no acid (PAC-NA), (**b**) No propolis and acid treated (PAC-A), and (**c**) Propolis and acid treated (MPAC-A).

Pull-out test showed that the force required to remove a PAC cylinder from the tooth cavity is comprehensively higher than in the case of the shear bond test. However, this test allowed us to compare the different treatments on the inner tooth surface, i.e., the force required to remove the PAC after acid treatment was 206.6 N, and that required after propolis treatment was only 57.1 N, being even lower than the force required for PAC removal without propolis and acid etching (151.4 N). Figure 7 shows the type of failure during this test.

The adhesion strength of MPAC is summarized in Table 3. For comparison purposes, the force reached during the shear adhesion test is reported along with the detaching force during the pull-out test. No experiment was conducted for 25MPAC, as the aim of the experiment was to assess propolis treatment on the dentine surface, not for propolis incorporated into PAC.

## 4. Discussion

Propolis has shown various degrees of antibacterial activity, depending on the source, concentration, solvent, or vehicle used, the bacterial strain tested, and the type of dental material used for modification. In our study, it was shown that 20% ethanolic solutions of propolis added to a PAC exhibited mild antibacterial activity against *S*. *mutans*, a Gram-positive bacterium, both as an extract and by means of disc-diffusion assay. When comparing this activity to traditional antibiotics, Hatunoglu et al. reported 3.9 µg/mL and 7.8 µg/mL MIC antibiotic values against *S*. *mutans* for Ampicillin and Gentamicin, respectively, while a value of 15.7 µg/mL for their 10 wt% ethanolic extracts of propolis was reported [27]. Another study reported a MIC of 60 µg/mL and 0.5 µg/mL for Penicillin and Chlorhexidine (0.2%), respectively [42]. This bacterium is involved in secondary caries, but it is also reported that its activity depends on the presence of *S*. *sanguinis* [43]. In this regard, the bactericidal effect of propolis has been attributed to the presence of flavonoids being more effective against *Streptococcus salivarius* rather than *Streptococcus mutans* [35]. In this line of thought, we have reported that the major components of propolis were pinocembrin, pinobanksin-3-O-acetate, and pinobanksin-3-O-propionate, which can be partly responsible for its mild antibacterial behavior [32].

Mild antibacterial activity, however, can be compensated for by antioxidant activity due to flavonoid and phenol content, in addition to radical scavenging activity. Furthermore, previous results from our group demonstrated the in vitro anti-inflammatory activity of propolis, as the levels of pro-inflammatory IL-1β, IL-6, and TNF-α were low, while the levels of IL-10 and IL-4 were high. Overall, the clinical performance of the propolis MPAC will depend not only on its antibacterial properties, but also on its antioxidant activity and anti-inflammatory properties [32]. In addition, it has been reported that propolis is used for the treatment of candidiasis, acute necrotizing ulcerative gingivitis, gingivitis, periodontitis, and pulpitis in dentistry, and there are reports regarding the antibacterial effects of propolis on methicillin-resistant Staphylococcus aureus. It also exhibits antifungal effects comparable to those of Nystatin, and displays the antimicrobial effects of propolis on anaerobic oral bacteria, such as *S*. *aureus*, *Actinobacillus*, and oral pathogenic micro-organisms such as *Streptococcus salivarius*, *Streptococcus sanguinis*, *Streptococcus mitis*, *Candida albicans*, *Streptococcus mutans,* and *Shigella* [44].

As propolis exhibited mild antibacterial behavior against *S*. *mutans*, only high propolis concentrations in PAC were studied, i.e., 25 µL and 50 µL. However, Table 2 clearly shows the deleterious effect on mechanical properties when increasing the concentration of propolis in PAC. The compressive and flexural properties were reduced to levels below the requirements of ISO 9917 for restorative materials. However, those formulations prepared with 25 µL of propolis were close to the required value of 100 MPa for compression in restorative materials but were suitable for base/lining cement and, therefore, are solely used for adhesion tests. The low mechanical properties observed can be explained due to curing kinetics, which is affected by the presence of various components in propolis, such as flavonoids, with the ability to form chelates, which, in turn, can sequester available divalent (calcium) or trivalent (aluminum) ions [45].

The adhesion of PAC to the tooth is the result of two phenomena: micromechanical interlocking and chemical bonding [8]. In this study, two types of adhesion tests were evaluated. In the first case, the traditional shear test was used to assess the propolis effect. The cured cylinders that contained propolis exhibited only 16.6 N of shear force on the unmodified dentine surface, but when dentine was either propolis- or non-propolis-treated and acid etched, the shear force increased up to 19.4 N (equivalent to a shear bond strength of 1.71 ± 0.74 MPa) and 19.3 N (equivalent to a shear bond strength of 1.70 ± 0.53 MPa), respectively, suggesting that the microroughness promoted adhesion [46]. The maximum shear bond force could be achieved via the direct placement of the untreated PAC cylinder onto the acid-treated dentine surface, reaching up to 38 N (3.72 ± 0.65 MPa). In contrast, the minimum shear bond force was 7.02 ± 3.0 N (0.62 ± 0.27 MPa) from the non-propolis-treated dentin and nonacid-treated dentin unmodified glass ionomer cements (PAC-NA), suggesting that the surface macroroughness achieved by polishing alone was not enough to adhere to PAC [47]. During debonding, a mixed type of adhesive failure was noted, i.e., adhesive and cohesive. When no propolis and no acid were used, the responsible factor for the mechanical interlocking is the surface roughness achieved after polishing, Figure 6a. When the dentine surface was acid-treated only, additional changes in surface topography were included, the dentine tubules were exposed, and adhesion was promoted, as shown in Figure 6b. However, the presence of propolis and no acid treatment did not allow PAC adhesion, probably due to the nonpolar compounds found in propolis. This can be alleviated by using acid etching, as shown in Figure 6c. Therefore, it is clear that any PAC modification (not only with propolis) must consider the presence of nonpolar compounds, as they will affect not only their adhesion and the type of failure but also the surface roughness accomplished by polishing (number of abrasive paper or grit size) and acid etching (type of acid, exposure time, etc.). However, other factors, such as dentine/enamel quality and the tubular density used, can alter the outcome [16,48].

In the second variation of the conventional shear test, the effect of propolis was studied by means of the pull-out test. From Table 3, no propolis and no acid treatment resulted in a high detaching force (151.4 N), as more area was exposed to the PAC when the roughness was introduced by drilling. When the sample is propolis-treated but not acid-treated, the detaching force was reduced by up to 57 N, i.e., the nonpolar compounds of propolis are covering the internal surface, limiting the diffusion of the PAC on the dentine [49]. Once again, when the sample is acid-treated only and no propolis is used, the highest detaching force is achieved (206 N). The higher force reached during this type of test in comparison to the conventional shear bond test responds to a higher uncontrolled roughness achieved during drilling and due to the higher exposed area [50]. Even when this type of test is not customary in all laboratories, it provides a more realistic approach to estimating PAC-tooth adhesion, as it involves a higher dentin area. Some of the limitations include the proper alignment of the metallic root, the formation of a true cylindrical cavity in the teeth (different from molars), and the achievement of the same roughness in the internal surface, among others.

When comparing the two sets of results, there is no apparent correlation, and this only confirms that the use of acid etching is enough to achieve the maximum adhesive strength [51]. However, the presence of propolis in PAC can compromise curing and adhesion. When our results are compared with other works, large variations are observed. For example, the tensile bond strength of Fuji IX to dentin was reported to be equal to 3.08 MPa, which is lower than the reported 5.0 MPa for adhesion to enamel [16]. Even when tensile and shear bond strength tend to be very similar, the reported values here are, in general, lower than those reported for the microshear tests that made use of smaller areas (1 mm^2^) [38]. Furthermore, Tedesco has reported that the microshear bond strength of Fuji IX depended on the density of the tubules and their location, i.e., from 3.20 MPa in occlusal deep dentin to 4.70 MPa in superficial occlusal dentin [48].

## 5. Conclusions

The addition of propolis to the PAC mixture had a response against a strain characteristic of the oral cavity. Despite the reduction in both compressive and bending strengths, the adhesion showed no statistical difference at shear bond strength (*p* < 0.05); thus, this material can be used in conditions where the affected property requirements are not critical, i.e., as with the cavity liner to the isolate surfaces near the pulp tissue when the atraumatic restorative technique is used, specifically due to the reported antibacterial effect against *S*. *mutans*.

Furthermore, the anti-inflammatory properties of propolis incorporated into this modified material could interact with the fluid of the dentinal tubules for the treatment of reversible pulpitis, and these formulations can be explored for pulpar treatments.

## Figures and Tables

**Table 1:** Compositions and abbreviations used for experimental samples.

Sample	Preparation
**PAC**	Unmodified material
**25MPAC**	PAC + 25 µL of propolis
**50MPAC**	PAC + 50 µL of propolis
**MPAC-NA**	PAC + 10 µL without acid tissue conditioning
**MPAC-A**	PAC + 10 µL with acid tissue conditioning
**PAC-NA**	PAC without acid tissue conditioning
**PAC-A**	PAC with acid tissue conditioning
**FASP**	Fluoroaluminosilicate powder

**Table 2:** Mechanical properties of PAC and MPAC.

Sample	Compressive Properties			Bending Properties		
	EC (MPa)	σC (MPa) *	εC (%)	EF (GPa)	σF (MPa) *	εF (%)
**PAC**	89.44 ± 8.88	211.21 ± 8.83 ^a^	2.45 ± 0.33	19.72 ± 6.40	11.10 ± 1.71 ^a^	0.10 ± 0.02
**25MPAC**	34.7 ± 7.13	94.40 ± 9.62 ^b^	2.43 ± 0.26	7.12 ± 2.51	7.78 ± 1.41 ^b^	0.29 ± 0.092
**50MPAC**	20.4 ± 3.5	59.36 ± 2.45 ^b^	3.23 ± 0.15	3.53 ± 1.76	6.25 ± 1.85 ^b^	0.42 ± 0.16

MPa = megapascal, GPa = gigapascal, E = elastic modulus, σ = strength, ε_F_ = maximum deformation; * = one-way ANOVA *p* < 0.05; the groups not sharing letters (^a^ or ^b^) are statistically different, according to Bonferroni posthoc.

**Table 3:** Adhesion strength of PAC and MPAC.

Sample	Shear Force (N) *	Shear Strength (MPa) *	Pull-Out Force (N) *
PAC-A	19.3 ± 6.0 ^a^	1.70 ± 0.53 ^a^	206.6 ± 27.1 ^a^
PAC-NA	7.02 ± 3.0 ^b^	0.62 ± 0.27 ^b^	151.4 ± 92.4 ^a^
25MPAC	16.6 ± 2.4 ^a^	1.47 ±0.211 ^a^	-
MPAC-A	19.4 ± 8.4 ^a^	1.71 ± 0.74 ^a^	57.1 ± 12.5 ^b^

MPa = megapascal, N = Newtons, * = one-way ANOVA *p* < 0.05; groups not sharing letters (^a^ or ^b^) are statistically different, according to Bonferroni posthoc.

## Data Availability

The data presented in this study are available on request from the corresponding author.
